# Efficient aqueous remote loading of peptides in poly(lactic-co-glycolic acid)

**DOI:** 10.1038/s41467-022-30813-7

**Published:** 2022-06-08

**Authors:** Morgan B. Giles, Justin K. Y. Hong, Yayuan Liu, Jie Tang, Tinghui Li, Avital Beig, Anna Schwendeman, Steven P. Schwendeman

**Affiliations:** 1 Department of Pharmaceutical Sciences and the Biointerfaces Institute, University of Michigan, North Campus Research Complex, 2800 Plymouth Road, Ann Arbor, MI 48109 USA; 2 Department of Biomedical Engineering, University of Michigan, 2200 Bonisteel Blvd, Ann Arbor, MI 48109 USA

**Keywords:** Biopolymers, Pharmaceutics, Nanocomposites, Drug delivery

## Abstract

Poly(lactic-co-glycolic acid) (PLGA) long-acting release depots are effective for extending the duration of action of peptide drugs. We describe efficient organic-solvent-free remote encapsulation based on the capacity of common uncapped PLGA to bind and absorb into the polymer phase net positively charged peptides from aqueous solution after short exposure at modest temperature. Leuprolide encapsulated by this approach in low-molecular-weight PLGA 75/25 microspheres slowly and continuously released peptide for over 56 days in vitro and suppressed testosterone production in rats in an equivalent manner as the 1-month Lupron Depot®. The technique is generalizable to encapsulate a number of net cationic peptides of various size, including octreotide, with competitive loading and encapsulation efficiencies to traditional methods. In certain cases, in vitro and in vivo performance of remote-loaded PLGA microspheres exceeded that relative to marketed products. Remote absorption encapsulation further removes the need for a critical organic solvent removal step after encapsulation, allowing for simple and cost-effective sterilization of the drug-free microspheres before encapsulation of the peptide.

## Abstract (web summary)

Encapsulation of bioactive peptides in slow-release particles is complex and relies on organic solvents. Here, the authors absorb peptides in a polymer phase from water, creating a simple low-cost encapsulation process in a class of polymer depot.

## Introduction

Peptide drugs with molecular weights from a few hundred to a few thousand Daltons form a unique class of drugs with both unique mechanisms of the pharmacological action and physical-chemical properties^1^. Once beyond a few amino acids peptides are often difficult to deliver to the body owing to poor bioavailability by noninvasive routes of drug administration and short serum half-lives^2^. Two common methods to improve systemic exposure and minimize injections of peptides include half-life extension via covalent modification and microencapsulation in biodegradable PLGA systems^3,4^. Microencapsulation has the advantages of much longer intervals between injections (>weeks to months) and no new active pharmaceutical ingredient (API) needs to be developed, thus reducing regulatory obstacles.

Conventional microencapsulation approaches to manufacture PLGA microspheres include solvent evaporation, coacervation, and spray-drying. In each of these methods, the API is combined with PLGA dissolved in an organic solvent before forming microspheres. This combination creates a number of undesirable issues: (a) the peptide-loaded microspheres most often cannot be terminally sterilized, thus requiring expensive aseptic processing with organic solvents and numerous unit operations; (b) the API is expensive, and therefore it is undesired to discard poorly formed drug-polymer microspheres (tiny fines, large aggregated microspheres, or debris on mixing equipment) and yields can be far less than 100%; (c) the complexity of unit operations and components necessary to form microspheres can be problematic to scale-up to large-scale manufacturing; (d) some products have one or more residual organic solvents, which pose challenges to storage stability of the final products;^5^ (e) there is little opportunity to manipulate the polymer structure once the peptide-PLGA matrix is formed, limiting the ability to engineer release kinetics^6,7^; and (f) mixing organic solvent/water mixtures in the presence of peptides, particularly with higher-order structure, can be detrimental to drug stability^8^.

Mechanistic analysis of peptide/PLGA interactions revealed a plausible solution to many of the above difficulties. Recently we discovered that two of the peptides currently used in commercial PLGA long-acting release depots (LARs), leuprolide and octreotide, long known to bind to the surface of uncapped PLGA (PLGA-COOH), can in fact be absorbed rapidly into the polymer phase of PLGA-COOH at high drug content with peptide desorption slow enough for potential long-term controlled release^9^. Organic solvent-free remote absorption encapsulation of aqueous peptide with drug-free PLGA-COOH particles could provide a number of clear advantages relative to conventional encapsulation approaches: (a) the process involves simple equilibration of peptides with the polymer particles and gentle mixing at modest temperature, bypassing complex unit operations and kinetic variations of particle formation and drying; (b) no organic solvent is used during encapsulation providing additional control over undesired residual solvents in the final product; (c) as the polymer particles are pre-formed before peptide exposure there is an opportunity to (i) sterilize the particles (e.g., via gamma irradiation) and (ii) pretest encapsulation on a small scale in order to reduce costs of goods associated with aseptic large-scale manufacture with organic solvents, and (iii) adjust the properties of the polymer particles (e.g., size distribution, porosity, residual organic solvent) before encapsulation for improved control of product performance; and (d) similar to use of remote loading liposomes^10^ and commercial transfecting agents^11,12^ sterile pre-formed microspheres are capable of encapsulating peptides at essentially 100% yields and with far less peptide needed compared to conventional encapsulation methods where even bench-scale procedures often require tens of milligrams of drug.

Remote loading based on absorption in the polymer phase is very different from a prior technique^13^ where macromolecular drugs, including peptides, were encapsulated by self-healing (or closing) of the aqueous pores in typical aliphatic ester end-capped PLGAs, which do not absorb peptides into the polymer phase^9,14^. Because of the minimal interaction between the peptide and end-capped PLGA^14^, encapsulation efficiency in porous PLGA is extremely low^14^ (e.g., <10%), and additional excipients need to be included in the formulation, such as dextran sulfate^8^ and aluminum adjuvants^14^ for trapping the drug in the pores of the polymer and MgCO_3_ for continuous release^15^ and protein stabilization^16^.

Here, we demonstrate a self-encapsulating PLGA microsphere, formulated from the same PLGA 75/25 employed in the well-known 1-month Lupron Depot®, which can be mixed with modest concentrations of aqueous peptide solutions to absorb peptides and form highly desirable peptide LARs. These formulations are capable of encapsulating a number of peptides at high efficiency and high drug loading, and then releasing the drug continuously with an acceptable initial burst release in vitro and in vivo.

## Results and discussion

**Fig. 1:**
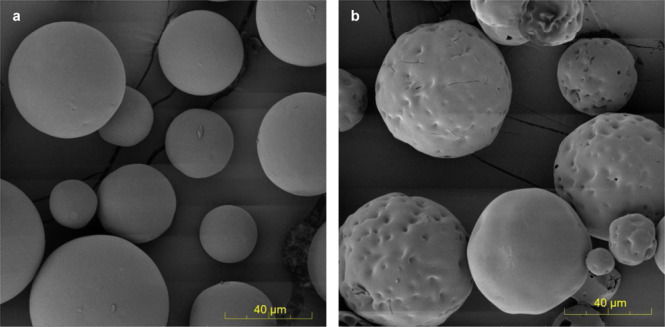
Scanning electron micrographs images of PLGA microspheres. Representative micrographs of (**a**) blank PLGA microspheres and (**b**) the blank microspheres remote-loaded with octreotide (*n* = 2 independent experiments).

To demonstrate the approach, we mixed sieved drug-free PLGA 75/25 microspheres (20–63 μm) with and without prior gamma irradiation at high microsphere concentration (180–240 mg/mL) with leuprolide, octreotide, and several other peptides (20 mg/mL) in a neutral pH HEPES buffer solution under mild agitation for 24 h at 37 °C (Fig. 1 and Supplementary Fig. 1).

**Fig. 2:**
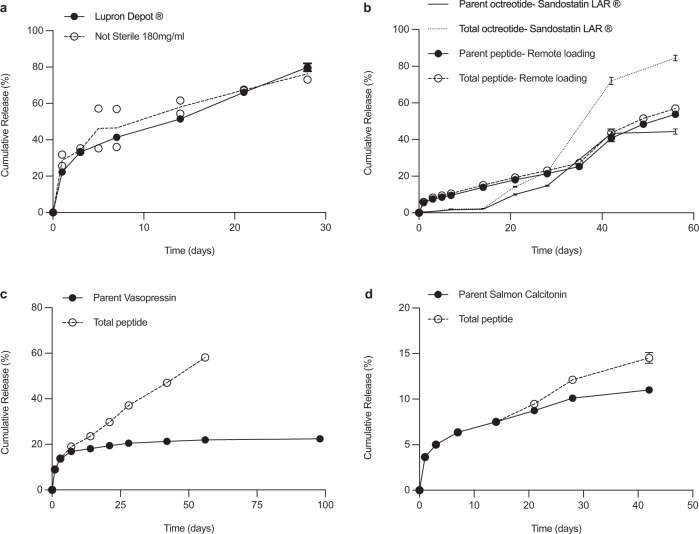
In vitro peptide release from remote loaded microspheres. Cumulative in vitro drug release from remote-loaded PLGA microspheres for (**a**) leuprolide compared to Lupron Depot®, NS (**b**) octreotide compared to Sandostatin LAR®, two-parent octreotide groups were significantly different on day 1, 3, 7, 14, 21, 28, 56, and two total octreotide groups were significantly different on day 1, 3, 7, 14, 21, 42, 56, (*p* < 0.05). **c** vasopressin, and **d** salmon calcitonin. Data are presented as mean values ± SEM (*n* = 3 independent experiments), except for leuprolide remote loading group (Not Sterile 180 mg/mL) in panel **a** was presented as mean with two individual data (*n* = 2 independent experiments). All remote-loaded microspheres were from non-sterile blank PLGA and from 180 mg/mL peptide solution. Parent and total peptide release listed for specific peptides not fully stable in the polymer. Sandostatin LAR® control data from Beig et al.^23^.

Microspheres were collected by centrifugation, washed, and lyophilized. All microspheres were suspendable with retention of near-spherical shape after peptide loading. For leuprolide, the microspheres loaded 6.63 ± 0.10 to 9.37 ± 0.03% w/w peptide at 66–94% efficiency (Supplementary Table 2) and slowly released leuprolide over 4 weeks in a PBS buffer in a similar manner, and with comparable burst release after 1 day (28.8–34.3%) (Supplementary Fig. 3) as the 1-month Lupron Depot® (Fig. 2a and Supplementary Fig. 3). Remote-loaded microspheres exhibited some small cracks (Fig. 1b and Supplementary Fig. 1c), likely owing to polymer rearrangement to accommodate the loaded peptide and the strong neutral pH buffer in the loading media, although this feature did not adversely affect microsphere performance (see below).

**Fig. 3:**
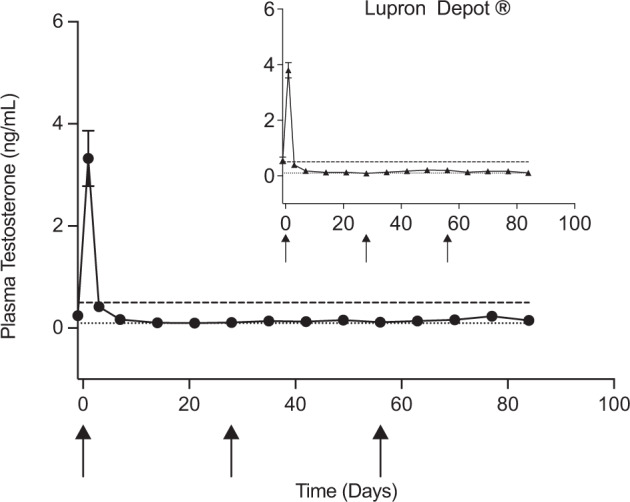
In vivo performance of remote loaded microspheres compared to commercial gold standard. In vivo plasma testosterone levels of male Sprague-Dawley rats administered 3 monthly doses of remote-loaded leuprolide PLGA microspheres compared to equivalent 1-month Lupron Depot® administration (inset), NS. Dashed line indicates castration level (0.5 ng/mL). Dotted line indicates analysis limit of quantitation (0.1 ng/mL). Data are presented as mean values ± SEM (*n* = 6 rats). Arrow indicates days of dosing. Remote-loaded microspheres were from 240 mg/mL leuprolide in PLGA microspheres previously exposed to gamma irradiation.

The leuprolide/PLGA microspheres were also administered to rats every 4 weeks for 3 months and compared for testosterone suppression relative to the Lupron Depot®. The testosterone plasma levels in animals receiving the remote-loaded formulation were not significantly different from that of the 1-month Lupron Depot (*p* > 0.05). After stimulating the downregulation of the gonadotropin-releasing hormone receptor, the animals receiving each of the formulations displayed sustained castration (Fig. 3 and Supplementary Fig. 6). Injection of negative control groups, either soluble free peptides or drug-free PLGA particles, are incapable of sustaining plasma testosterone below castration levels (0.5 ng/mL)^9^ necessary to minimize signaling of hormone-dependent cancer cell growth^17^.

**Fig. 4:**
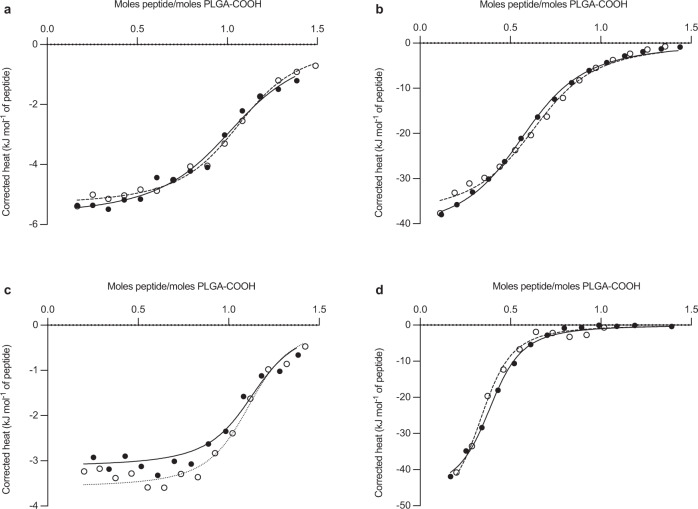
ITC thermograms of peptide binding to PLGA microspheres. ITC binding of leuprolide (**a**), octreotide (**b**), vasopressin (**c**), and salmon calcitonin (**d**) to 75/25 PLGA–COOH at 37 °C in DMSO. Data shown as heat released per molar ratio of peptide to –COOH groups of PLGA (*n* = 2 independent experiments).

To explore the generality of the approach we encapsulated several additional peptides under the same conditions as optimized with leuprolide and with the same drug-free microspheres. Strongly positively charged peptides (leuprolide, octreotide, vasopressin, salmon calcitonin and pramlintide) were all encapsulated at high efficiency (66–98%) and loading (6.6–9.8% w/w) in contrast with net negatively charged exenatide (23% efficiency, pI = 4.9)^18^ and mostly neutral protirelin (28% efficiency, single ionizable His residue) (Table 1, Supplementary Table 2). Consistent with these results, molar free energy of the peptide-PLGA-COOH binding determined in DMSO by isothermal titration calorimetry (ITC) (Fig. 4) was strongly favorable with stoichiometric coefficients of ~0.34–1.1 mole/mole peptide/PLGA-COOH group (Table 1).

**Table 1:** Peptide-PLGA binding parameters by isothermal titration calorimetry at 37 °C in DMSO and efficiency of remote loading in PLGA 75/25 microspheres by absorption at 1 day at 37 °C.

Peptide	Association constant (mM^−1^)	Stoichiometry		Δ *H* (kJ/mol)	−*T*Δ *S* (kJ/molK)	Δ *G* (kJ/mol)	Loading (%)	Encapsulation efficiency (%)
Leuprolide	22.6 ± 7.0	1.04 ± 0.01	−5.54 ± 0.01		−20.3 ± 1.1	−25.8 ± 0.9	9.64 ± 0.01	96.4 ± 0.02
Octreotide	14.3 ± 1.5	0.62 ± 0.04	−39.9 ± 2.7		15.2 ± 2.9	−24.7 ± 0.3	8.49 ± 0.01	84.9 ± 0.1
Vasopressin	56.4 ± 4.7	1.09 ± 0.01	−3.35 ± 0.33		−24.9 ± 0.2	−28.2 ± 0.3	8.00 ± 0.32	86.6 ± 3.4
Salmon Calcitonin	30.5 ± 4.0	0.34 ± 0.03	−49.2 ± 3.9		22.5 ± 4.0	−26.6 ± 0.2	8.17 ± 0.66	80.8 ± 5.0

Mean ± SEM (*n* = 2 independent experiments).

ITC curve fitting predicted binding as enthalpically favorable for peptides with ε-amino groups (Lys side chains in octreotide and calcitonin) indicative of strongly favorable intermolecular forces (e.g., a combination of ionic and H-bonding interactions)^19,20^. Entropically driven binding was determined for peptides with cationic Arg residues but no ε-amino groups (leuprolide and vasopressin), indicative of desolvation-driven interactions^21^. Cationic pramlintide, with significantly higher order structure^22^, also displayed high loading (9.8%) and binding to PLGA-COOH via ITC (Supplementary Fig. 5), although binding showed some anomalous behavior, prohibiting estimates of binding energies. Exenatide and protirelin displayed very low binding tendencies, as expected by their absence of positive charge (Supplementary Fig. 5). As with leuprolide, the 1-day remote absorption encapsulation procedure yielded PLGA microspheres that slowly released cationic peptides. Octreotide was released for >8 weeks after remote loading by desirable zero-order kinetics with minimal peptide instability, in contrast with the existing 1-month commercial Sandostatin LAR® (Fig. 2b), which releases by sigmoidal behavior with substantial peptide acylation and loss of parent drug. Vasopressin and salmon calcitonin were also slowly and continuously released (Fig. 2c, d), with the former showing behavior consistent with peptide damage^8^ in the PLGA carrier and the latter only releasing <20% parent peptide. The low level of salmon calcitonin release was found to be consistent with the instability of the drug in the release media (Supplementary Table 5). It is well known that certain peptides encapsulated in the commercial formulations display significant peptide instability (e.g., octreotide in Sandostatin LAR®^23^ and exenatide in Bydureon®^24^) and no attempt was made to introduce excipients to minimize these reactions. The presence of primary amino groups (as found in octreotide and calcitonin) is well established to lead to peptide acylation products consisting of adducts mostly from glycolic and lactic acid monomers and dimers^14,25^, which is consistent with a reduction in the bioavailability of the Sandostatin LAR® relative to the free peptide injection^26^. Leuprolide possesses a positive charge at neutral pH from its arginine residue without any primary amino groups and displays negligible instability in PLGA. Therefore, peptide release data (Fig. 2a and Supplementary Figs. 3 and 4) for leuprolide represents that of the parent drug.

**Fig. 5:**
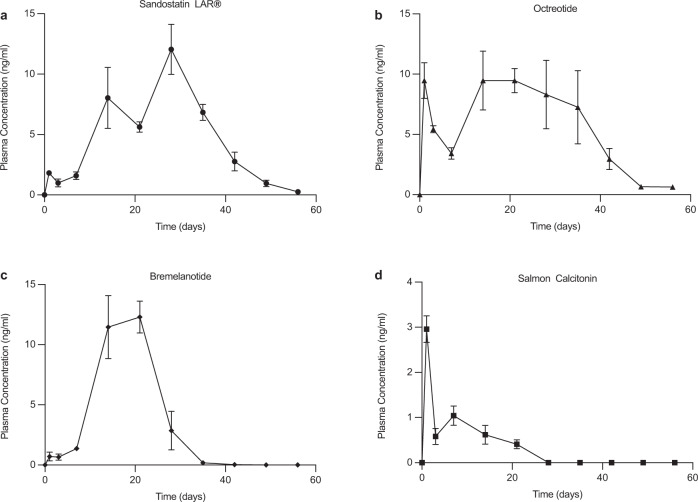
In vivo pharmacokinetic response to additional peptide-loaded microspheres. Pharmacokinetics after single subcutaneous injections of Sandostatin LAR® (**a**), and remote-loaded microspheres containing octreotide (**b**), bremelanotide (**c**), and salmon calcitonin (**d**) in male Sprague-Dawley rats. Rats in the octreotide and bremelanotide groups were administered a dose of 18 mg/kg. Rats in the salmon calcitonin group were dosed at 4 mg/kg. Data are presented as mean values ± SEM (*n* = 4 rats).

In order to verify that the slow-release behavior for the additional peptides recorded under in vitro conditions was also observed in vivo, we evaluated the pharmacokinetics in rats of octreotide and salmon calcitonin remote-loaded microspheres (Fig. 5). In addition, in place of vasopressin, which was also likely damaged by the erosion of PLGA, we encapsulated another cationic peptide, bremelanotide, a recently FDA-approved hormone indicated for generalized hypoactive sexual desire disorder (HSDD) in premenopausal women^27^. Remote-loaded microspheres of this latter peptide exhibited elevated encapsulation efficiency (83.7% ± 0.7) and a slightly higher burst than leuprolide or octreotide before steadily releasing for just over 4 weeks in vitro (Fig. 7). All peptide remote-loaded microspheres in this group showed a very slight increase in median size (D50 = 51–58 μm) relative to preformed peptide-free microspheres (D50 = 48 μm) (Supplementary Table 1), porous interiors (Supplementary Fig. 2) owing to the osmotic trehalose included in the preformed microsphere preparation procedure, and a strong increase in dry *T*_g_ (48–50 °C) relative to preformed microspheres (45 °C) due to the peptide-polymer binding (Supplementary Table 1)^28,29^.

As expected, single subcutaneous injections of microspheres in rats elevated the plasma concentration of each of the peptides for 3–6 weeks as predicted by the corresponding in vitro release kinetics. Most interesting was the systemic exposure of remote-loaded octreotide relative to commercial Sandostatin LAR®, which is formulated by the highly complex coacervation method in a glucose-star PLGA^30^. Relative to the commercial octreotide depot, the remote-loaded formulation exhibited more continuous plasma levels without a lag time and with as high or higher relative bioavailability (i.e., as indicated by the similar area under the plasma concentration-time curves (AUCs), 6460 ± 560 ng h/mL vs. 7640 ± 1040 ng h/mL, for Sandostatin LAR® and remote-loaded octreotide, respectively) (Fig. 5). There was no significant difference in AUCs for these two formulations. Both bremelanotide and salmon calcitonin have very short plasma half-lives in rats (<1 h)^31,32^, confirming that the remote-loaded formulations retain these peptides for weeks after administration. As each of the peptides possess different physical-chemical and biological properties, optimal in vitro and in vivo performance, as originally developed for leuprolide for the remote-loading formulation described here, may need to be adjusted depending on the desired release and stability behavior for the specific peptide of interest.

Few scalable microencapsulation methods for microsphere preparation are available^33,34^ and only a few new encapsulation techniques have been introduced with a clear path to commercialization. A spray congealing approach was developed to be compatible with fragile proteins such as human growth hormone and advanced to the FDA-approved Nutropin Depot^35,36^. However, this product had issues such as high initial burst release kinetics and was removed from the market several years after its approval^35,37^. In our absorption procedure, the initial burst release was modest and was dependent on the specific peptide encapsulated (Fig. 2). However, we did observe a ~40% drop in the initial burst of leuprolide (Supplementary Fig. 8) when loading in highly nonporous trehalose-free microspheres (Supplementary Fig. 2e) with very little effect on encapsulation efficiency (Supplementary Table 6). A more detailed comparison of remote-loaded microspheres relative to the commercial products, 1-month Lupron Depot® and Sandostatin LAR®, is also described in the Supplemental Material (Supplementary Tables 3 and 4).

As described above, we have previously introduced the concept of remote loading in aqueous solution^13,38^ by encapsulating large molecules including leuprolide and large proteins in porous aliphatic ester end-capped PLGAs, where pores are closed spontaneously by passive healing of the polymer with elevated temperature. This technique, devised to avoid micronization of proteins and their contact with organic solvent, currently requires a “trapping agent” to be placed inside the polymer pores to bind the entering drug to avoid low encapsulation efficiency^13^. The acid-capped polymer used here, owing to its more hydrophilic end groups, does not heal particularly well in comparison with ester-end-capped PLGAs^13,39^ and does not need a trapping agent as the carboxylic acid group binding initiates peptide entry into the polymer phase. As with self-healing encapsulation, remote loading by absorption requires that the loading temperature is held above the hydrated glass transition temperature (*T*_g_) of the polymer. For example, dry and hydrated Tg of PLGA 75/25 microspheres used for loading peptides is 46.3 ± 0.1 °C and 30.3 ± 0.4^ °^C, before loading, respectively, allowing for adequate mobility of the PLGA-COOH solid solution at 37 ^°^C for peptide entry^14^. This unique absorption and continuous release characteristic of low molecular weight PLGA-COOHs such as used in the Lupron Depot^40,41^ makes the current remote-loading method much simpler and readily scalable for use with small peptides. For large proteins where polymer-peptide interactions are not desired, the self-healing approach is warranted. It is noteworthy that remote loading is expected to be compatible with newer approaches toward producing PLGA microspheres that precisely control the size distribution^13,42^.

While the mechanism of absorption of peptides in PLGA-COOH is not fully understood, the available data strongly suggests that the cationic peptide forms a salt with surface carboxylate anions before moving into the highly swollen and mobile polymer phase as a peptide-PLGA ion pair. We previously verified the deep entry of peptides in the polymer phase by multiple orthogonal techniques, including stimulated Raman scattering and laser confocal fluorescence imagining, and serial microtome sectioning of thin PLGA films^9^. The peptide absorption was strongly inhibited or did not occur when the temperature was reduced to inhibit the mobility of the polymer, the pH of the solution was reduced to decrease the ionization of the PLGA carboxylic acid end groups, or by end-capping the PLGA that also removes polymer chain ionization^9^. The isothermal calorimetry binding of peptide and PLGA in DMSO (Table 1, Fig. 4 and Supplementary Fig. 5) also distinguished between peptides that were successfully remote loaded in the polymer and those that were not (Supplementary Table 2). The binding by this technique was found to be either enthalpically favored for amine-containing peptides and entropically favored for those peptides with positive charges resulting from arginine residues (Table 1).

In closing, the results here are significant in that we have demonstrated a remarkably easy, efficient, and scalable way of creating an effective 1-month LAR formulation for leuprolide and demonstrated the potential for other important peptides for controlled release (e.g., octreotide). Imagine the simple formulation of drug-free microspheres that are terminally sterilized and used to encapsulate peptides by a single aseptic aqueous mixing step. It is reasonable to expect that utilizing this approach could lead to a significantly reduced cost of goods relative to existing commercial formulations (e.g., Lupron Depot® and Sandostatin LAR®). Large scale encapsulation could be tested for a given microsphere batch on a small scale first to reduce the risk of large-scale batch failure and ensure final product performance. This simple encapsulation paradigm obviates (a) the complex steps necessary to prevent peptide loss during encapsulation by the solvent evaporation method^33,43^, (b) the use of oil and multiple organic solvents during the coacervation method^43,44^, and (c) low yields experienced by spray drying^44,45^. More work is needed to expand the potential of this encapsulation approach, namely, to determine (a) optimal drug-free microsphere formulation, encapsulation and sterilization conditions, (b) how to improve peptide instability in PLGA and related polymers, (c) how to apply the approach to peptides with neutral to negative net charge and other important charged biomacromolecules like nucleic acid drugs, and (d) how to incorporate additional functionality to the surface of the polymer before encapsulation. With these initiatives many more commercial peptide LARs can be envisioned in the future^46^.

## Methods

Our research complied with all relevant ethical regulations for animal testing and research. All protocols were approved by the University of Michigan (Ann Arbor, MI) Animal Care and Use Committees (IACUC) with protocol numbers PRO00007890 and PRO00009970.

### PLGA microsphere formulation

Drug-free PLGA microspheres were prepared by using a double emulsion solvent evaporation method. PLGA 75/25 13 kD Mw acid-capped (Wako, Japan), 1 g was dissolved in 1 mL methylene chloride. Once dissolved, 100 μl of a 500 mg/mL trehalose in ddH_2_O solution was added to the polymer solution to increase polymer porosity and then homogenized (VirTis Tempest I.Q.2) for 1 min at 10,000 rpm to create a water-in-oil (w/o) emulsion. Next, 4 mL of a 5% wt poly vinyl alcohol (PVA) solution was added to the emulsion, which was then vortexed (Scientific Industries Vortex Genie 2) for 1 min at maximum speed. The solution was then added to a 100 mL stirring bath of 0.5% PVA and stirred for 3 h to allow for solvent evaporation. After hardening, microspheres were washed with 1 L diH_2_O and sieved for size, 20–63 μm. Microspheres were lyophilized (Labconco FreeZone 2.5) at <0.1 mBar and stored, desiccated, at −20 °C until further use. To illustrate adjustment of initial burst release, the drug-free PLGA microspheres were prepared ± prior polymer purification and ± the trehalose porosigen, as described in Supplementary Material.

### Irradiation of microspheres

Preformed, drug-free microspheres were weighed out into glass ampules, ~100 mg per ampule, and sealed under vacuum. Sealed ampules were exposed to gamma irradiation at a dose of 1.8 Mrad and dose rate of 0.64 Mrad/h using a ^60^Co source. Radiation treatment was performed by the Oregon State University Radiation Center (Corvallis, OR).

### Remote loading of microspheres by peptide absorption

Microspheres with or without terminal sterilization were loaded by incubating 90 mg of microspheres in 0.1 M HEPES buffer pH 7.4 with 20 mg/mL dissolved peptide for 24 h at 37 °C with gentle rotational agitation. Loading solution volume was 0.5 mL or 0.375 mL for leuprolide giving respective 180 and 240 mg/mL initial microsphere concentrations. All other peptides were loaded at 180 mg/mL microspheres concentration solution under identical conditions. After incubation, microspheres were centrifuged for 5 min at 7012 × *g* and the supernatant was removed. Loaded microspheres were washed three times with 1 mL of diH_2_O. Loaded microspheres were then freeze dried and stored at −20 °C.

### UPLC analysis of peptides

Leuprolide concentration in the solution was analyzed by UPLC (Waters, Parsippany, NJ). Prior to analysis solutions were syringe filtered (0.45 μm). Samples were run on a C18 (Aquity BEH C18, 0.7 μm, 2.1 × 100 mm) column. The mobile phases were composed of (A) acetonitrile containing 0.1% trifluoroacetic acid (TFA) and (B) diH_2_0 containing 0.1% TFA. The gradient condition for leuprolide started from 25% A:75% B and increased to 35% A:65% diH_2_O + TFA B over 3.5 min at a flow rate of 0.5 mL/min. The same gradient method was used to analyze octreotide samples at a flow rate of 0.4 mL/min for 4 min. Vasopressin was analyzed using an isocratic method of 20% A:80% B at 0.4 mL/min over 2 min. Salmon calcitonin samples were also run isocratically at 37% A:63% B for 1.8 min at 0.5 mL/min. Exenatide samples were run on a gradient of 25% A:75% B to 90% A:B over 4 min at a flow rate of 0.5 mL/min. All peptides were analyzed on the same column and detected by UV at 215 and 280 nm.

### Evaluation of peptide loading and encapsulation efficiency by mass loss

Mass loss was used to determine leuprolide, vasopressin, salmon calcitonin, octreotide, protirelin, and exenatide loading. To accomplish this, the supernatant was removed after loading and wash solutions were analyzed by UPLC (Acquity H-Class, Waters) as detailed above. The volume of loading solution, which changes during encapsulation with a high concentration of suspended microspheres, was determined after encapsulation by centrifuging (5 min, 1753 × *g*) the particle suspension and then removing 200 μL of supernatant. Particles were then washed by adding 1 mL of diH_2_O to the particles then centrifuged again and 900 μL of supernatant removed. The wash step was repeated two more time for a total of three wash steps. Peptide loading is calculated from the weight ratio of peptide encapsulated to total microsphere weight (w/w %). Encapsulation efficiency is the ratio of actual peptide loaded to theoretical peptide loading (total peptide in solution/ (total peptide and polymer added)).

### Evaluation of peptide loading and encapsulation efficiency by nitrogen analysis

Total nitrogen analysis was used to determine the loading of octreotide, exenatide, protirelin, pramlintide, salmon calcitonin, vasopressin, and bremelanotide by using a modified automated Dumas technique^46^. Briefly, ~2 mg of loaded microspheres were aliquoted into tin pans, in triplicate, then crimped to remove excess air prior to analysis. Samples were run on a Leco TrueSpec® Micro CHN (LECO, USA). The instrument was first blanked without samples or tins to establish atmospheric baselines. Using USP grade EDTA the percent of carbon, hydrogen, and nitrogen were verified to be within the anticipated range and these values were set as standards. Lyophilized peptide standards were run to verify the percent nitrogen in the peptide and set a peptide factor. Microsphere samples were then dropped into a combustion chamber at 1050 °C, which converts all sample nitrogen to nitrogen gas, which is then quantified by a thermal conductivity cell. Peptide content was determined by multiplying the nitrogen mass by the peptide factor after first subtracting the nitrogen mass from negative controls (unloaded microspheres).

### Evaluation of PLGA glass transition temperature

The dry and hydrated glass transition temperature (*T*_g_) of 75/25 PLGA microspheres was measured by differential scanning calorimetry (DSC) analysis (Discovery DSC, TA Instruments, New Castle, DE). Drug-free irradiated and non-irradiated microspheres (4–6 mg) were weighed and added to non-hermetic pans. For hydrated measurements, drug-free irradiated and non-irradiated microspheres (10–12 mg) were weighed and suspended in 200 μL of 0.1 M HEPES solution (pH 7.4) at 37 °C for 24 h on a rotator. After incubation, microspheres were centrifuged, and the supernatant was removed to create a slurry. Both dry and hydrated samples underwent a heat/cool/heat cycle 5–90 °C, at 1 °C/min. DSC results from the second heat cycle were used for analysis.

### Evaluation of microsphere size distribution and morphology

The volume-weighted particles size and size distribution of the blank and peptide-loaded microspheres were determined using a Malvern Mastersizer 2000 (Malvern Instruments Ltd., Worcestershire, U.K.). Dry microspheres were suspended in ddH_2_O at a concentration of 50 mg/mL and then added dropwise to instrument sample dispersion unit until the obscuration reached 5−10%. Three measurements were performed for each sample at a stirring speed of 2500 rpm using a material refractive index of 1.59 and an absorbance of 0.01. The *D*(0.1), *D*(0.5), *D*(0.9) and span of microspheres were recorded to determine the particle sizes and distribution. The span of the microsphere size distribution was calculated by:

1 $${{{{{\rm{Span}}}}}}=\,\frac{D\left(0.9\right)-D\left(0.1\right)}{D\left(0.5\right)}$$

where *D*(0.1), *D*(0.5), *D*(0.9) are the microsphere diameters measured at the 10th, 50th and 90th, percentiles of undersized microparticles, respectively.

The morphology of microspheres was examined by using TESCAN MIRA3 FEG scanning electron microscope (SEM) (Kohoutovice, Czech Republic). The microspheres were mounted on brass stubs using double-sided carbon adhesive tape. To analyze the internal morphology, samples were fractured with a razor blade. Samples on the stubs were then sputter coated with a thin layer of gold for 70 s at 18 mA under vacuum. Images were taken with a 5 kV excitation voltage.

### Evaluation of in vitro release of peptide-loaded microspheres

Leuprolide release from irradiated and non-irradiated microspheres loaded at 180 mg/mL and 240 mg/mL peptide solution concentration was measured in vitro by determining the percent remaining in microspheres, as was done by Ogawa et al.^29^. Briefly, 10 mg of loaded microspheres were weighted out, in duplicate, for each time point to be tested (Day 1, 3, 5, 7, 14, 21, 28), 14 samples total and incubated with 1 mL of phosphate-buffered saline + 0.02% Tween 80 (PBST) with mild agitation at 37 °C. At each time point all samples were centrifuged for 5 min at 7012 × *g* and 900 µL of the release buffer was removed to ensure that no particles were collected. Samples for Day 1 were then stopped and dried for 24–48 h at 25 °C. For all remaining samples, for days 3–28, 900 µL of the removed release media was replaced with fresh PBST and were incubated until the next time point. This process was repeated at each time point.

Once samples were stopped and dried on their specific sampling day approximately 5 mg of microspheres were weighed out into an Eppendorf tube for analysis of percent peptide remaining by two-phase extraction as described in the section “Evaluation of peptide remaining by two-phase extraction” below. For all other peptides the parent and degraded peptide peaks in the release media were quantified by UPLC analysis as described above.

### Evaluation of peptide remaining by two-phase extraction

Extraction of peptides in microspheres was used to determine the amount of peptides remaining after incubation for leuprolide release as well as both leuprolide and octreotide encapsulation during loading. Briefly, ~5 mg of loaded microspheres were dissolved in 1 mL methylene chloride and 2 mL of 1 M sodium acetate (pH 4) was added. The two-phase mixture was vortexed for 1 min then centrifuged for 4 min at 1753 × *g* and 1.5 mL of the upper aqueous phase was removed. The removed 1.5 mL was replaced with a fresh 1.5 mL of sodium acetate and the process was repeated four more times for a total of five extractions. After the 5th extraction, 1.5 mL of 1 M sodium acetate + 1 M sodium chloride (pH 4) was added. This process of vortexing then centrifugation was repeated 6 times resulting in 11 total extractions, which were analyzed by UPLC^9^.

### Evaluation of efficacy of leuprolide-loaded microspheres in rats

The efficacy of leuprolide-loaded microspheres for testosterone suppression was tested in male Sprague-Dawley rats (7-week old) and compared to commercial 1-month Lupron Depot^®^. Both non-irradiated and irradiated microspheres were injected into rats at a dose of 100 µg/kg/day based on drug loading by mass loss from solution. Microspheres were suspended in an injection vehicle of 0.5% low viscosity carboxy methylcellulose (CMC), 0.1% w/v Tween 80 and 5% D-mannitol and were administered subcutaneously. Lupron Depot^®^ was reconstituted and administered according to the package insert^47^. Blood was sampled from the jugular vein on days 1, 3, 7, and weekly thereafter to determine plasma testosterone levels. Whole blood samples were centrifuged for 10 min at 1753 × *g* (Eppendorf 5810R) to separate the plasma. The plasma was then removed and stored at −80 °C until analysis. Plasma testosterone samples were analyzed, in duplicate, by RIA analysis at the University of Pennsylvania (RIA Biomarkers Core, Penn Diabetes Center).

Animal studies were conducted according to the University of Michigan Committee on Use and Care of Animals (IACUC) protocol numbers PRO00007890 and PRO00009970.

### Evaluation of pharmacokinetics of peptide-loaded microspheres in rats

The pharmacokinetics studies of peptide-loaded microspheres were performed in male Sprague-Dawley rats. Sandostatin LAR® and peptide-loaded microspheres were suspended in 1 mL 0.5 % low viscosity carboxy methyl cellulose, 0.1 % w/v Tween 80 and 5 % D-mannitol and subcutaneously injected into the back of rats (*n*  = 4, 18 mg/kg for octreotide and bremelanotide, and 4 mg/kg for salmon calcitonin). Blood samples were drawn from the jugular vein at predetermined time points (Day 1, 3, 7, 14, 21, 28, 35, 42, 49, 56). The blood samples were centrifuged at 1753 × *g* for 10 min, and the plasma was stored at −80 °C until analysis.

The quantification of octreotide and bremelanotide in rat plasma was performed by UPLC-MS analysis. Briefly, 100 μL of plasma was mixed with 10 μL of internal standard (100 ng/mL of leuprolide in 25% acetonitrile) and 10 μL of acetic acid and was vortexed for 1 min. 300 μL of acetonitrile containing 0.1% formic acid was then added and the mixture was vortexed for another 1 min for protein precipitation. The samples were centrifuged at 15,777 × *g* for 10 min and the supernatant was transferred to a clean tube followed by evaporation under nitrogen flow. The residue was reconstituted in 100 μL of 25% acetonitrile containing 0.1% formic acid, centrifuged at 15,777 × *g* for 10 min, and the supernatant was injected into the LC-MS system. The standard curve was prepared by adding octreotide or bremelanotide solution (dissolved in 25% acetonitrile) into 100 μL of blank rat plasma, and the standard curve samples were processed using the same method as above for testing samples. All the samples were analyzed by LC-MS (Shimadzu HPLC system and AB Sciex QTrap 5500 mass spectrometer) using a Waters Xbridge® C18 column (2.1 × 50 mm, 3.5 μm). The mobile phase consisted of diH_2_O containing 0.1% formic acid (A) and acetonitrile containing 0.1% formic acid (B), and the flow rate was 0.4 mL/min. The gradient elution started from 15% B, changed to 40% B within 1.8 min and held for 0.7 min, then changed to 95%B within 0.1 min and held for 0.9 min, finally returning to 15%B within 0.1 min and maintained for 1.9 min for equilibration.

The concentration of salmon calcitonin in rat plasma was determined using an ELISA kit (Salmon Calcitonin ELISA Kit, abx150375, Abbexa). The assay was performed by the Cancer Center Immunology Core at the University of Michigan following the ELISA kit manual. The PK parameters were calculated with MATLAB using a non-compartmental model.

### Acid end-group content of PLGA

Acid content was determined similarly as described by Mehta et al.^48^. Briefly, 150 mg of 75/25 PLGA microspheres were dissolved in 20 mL of a 1:1 tetrahydrofuran:acetone (THF: acetone) mixture. Approximately 7 drops of 0.5 wt % phenolphthalein was added to the solution of dissolved microspheres. The solution was then titrated with 0.01 M methanolic KOH until the solution was observed to maintain a slight pink color through the solution. The acid end-group content was then calculated as:

2 $${{{{{\rm{Acid}}}}}}\; {{{{{\rm{end}}}}}}\; {{{{{\rm{group}}}}}}\; {{{{{\rm{content}}}}}}=\,\frac{\left({{{{{\rm{Volume}}}}}}\; {{{{{\rm{of}}}}}}\; {{{{{\rm{titrant}}}}}}\right)* \left(0.01{{{{{\rm{M}}}}}}\; {{{{{\rm{KOH}}}}}}\right)}{{{{{{\rm{mass}}}}}}\; {{{{{\rm{of}}}}}}\; {{{{{\rm{PLGA}}}}}}\; {{{{{\rm{microspheres}}}}}}}$$

### Isothermal titration calorimetry of peptide-PLGA interactions

Peptide salts were dissolved in dimethylsulfoxide (DMSO) to prepare a final peptide solution of 7.88 mM. PLGA 75/25 polymer was dissolved in DMSO for a 1.68 mM polymer solution based on the acid content of PLGA (0.261 ± 0.004 mmol/g PLGA, *n* = 3), as previously described^49^. Prepared peptide and polymer solutions and DMSO were degassed 10 min prior to NanoITC (TA Instruments, New Castle, DE) calorimetry studies to eliminate air bubbles. Degassed DMSO was added to the reference cell (300 μL) and the polymer solution (300 μL) was added to the sample cell. The ITC syringe was filled with 50 μL of the degassed peptide solution. The polymer solution was titrated with 15 injections of 2.5 μL peptide solution/injection with an injection interval of 300 s and constant stirring of 350 rpm. Heat produced by peptide dilution was evaluated by a control experiment, titrating peptide solution into DMSO under the same experimental conditions. The interaction heat for each injection was calculated by correcting the heats from the peptide dilution. The resulting corrected injection heats were plotted as a function of the molar ratio of peptides to the acid number of PLGA, fitted with a model for one-site independent model and analyzed with a nonlinear least-squares minimization algorithm by using the NanoAnalysis software, version 3.7.5 (TA Instruments, New Castle, DE). From the data fit the molar enthalpy change, reaction stoichiometry and peptide dissociation constant were obtained. From model fits, the molar entropy and Gibbs free energy changes and peptide association constant were also calculated^50^.

### Scanning electron micrographs

Microsphere surface and internal morphology of particles shown in Fig. 1a, b, Supplementary Fig. 1a–d, and Supplementary Fig. 2a–f were imaged by adhering a representative sample (~0.1 mg of particles) to an SEM stub then sputter coating particles for 90 s prior to imaging (AMRAY 1910 Field Emission Scanning Electron Microscope) with a gun voltage of 5 kV.

### Statistical analysis

All of the results presented in this study are Mean ± SEM. Statistical comparison of in vitro release from peptide remote loaded microspheres and commercial microspheres at each time point was performed by a two-tailed multiple unpaired *T*-test with Welch’s correction assuming *p* < 0.05 for statistical significance. Statistical comparison of plasma testosterone levels for Lupron Depot® and remote-loaded leuprolide was performed by a two-tailed unpaired *T*-test with Welch’s correction assuming *p* < 0.05 for statistical significance. Statistical comparison of the area under the plasma concentration-time curve of Sandostatin LAR® and remote-loaded octreotide was performed by two-tailed unpaired *T*-test assuming *p* < 0.05 for statistical significance.

### Reporting summary

Further information on research design is available in the Nature Research Reporting Summary linked to this article.

## Supplementary information

Supplementary Information

Reporting Summary

## Data Availability

The authors declare that all data supporting the results in the study are available within the paper and its supplementary information. Additionally, data is available on FigShare (https://figshare.com/account/home#/projects/81131) and can be provided upon request.
