# Supplementary material for: Efficient aqueous remote loading of peptides in poly(lactic-co-glycolic acid)
Source: Nat Commun. 2022 Jun 8;13:3282. doi: 10.1038/s41467-022-30813-7 (PMC9177552; doi:10.1038/s41467-022-30813-7)
Supplement: Supplementary file 1 — Supplementary Information [file 41467_2022_30813_MOESM1_ESM.pdf]

Supplementary Information:

Efficient aqueous remote loading of peptides in poly(lactic-co-glycolic acid)

S. P. Schwendeman *et al.*

Supplementary Notes

The remote loading method allows for microsphere formulation and optimization prior to peptide encapsulation. Very few pores were visible on the surface (Supplementary Fig. 1) of preformed drug-free microspheres despite the presence of a porosigen (trehalose) in the formulation. Microspheres prepared in this way tend to have significant porosity, as measured by mercury intrusion [13,51,52] to facilitate rapid mass transfer and peptide encapsulation. Gamma irradiation did not significantly affect the surface morphology of the microspheres (Supplementary Fig. 1). However, microspheres lost their smooth surface after peptide loading (Supplementary Fig. 1) as the peptide forms the salt with the PLGA carboxylic acid end groups.

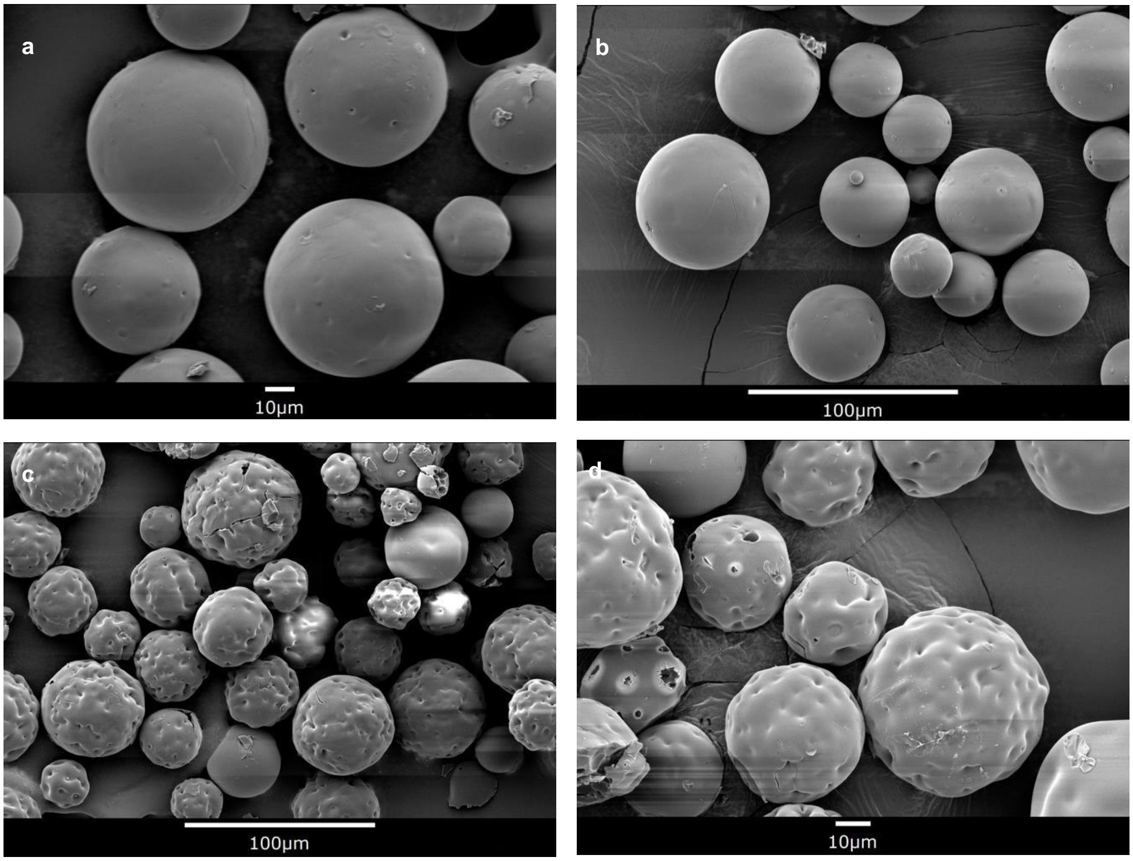

Supplementary Figure 1. Effect of gamma irradiation and peptide loading on particle surface morphology. Representative scanning electron micrographs of (a) PLGA microspheres prior to

gamma irradiation, (b) after gamma irradiation, (c) non-irradiated leuprolide-loaded PLGA microspheres loaded at 180 mg/mL, and (d) non-irradiated leuprolide-loaded PLGA microspheres loaded at 240 mg/mL. (n > 3 independent experiments).

After incubation with peptide solutions microspheres retain their internal porosity as seen when comparing a particle prior to peptide loading to particles loaded with leuprolide, octreotide, salmon calcitonin and bremelanotide (Supplementary Fig. 2).

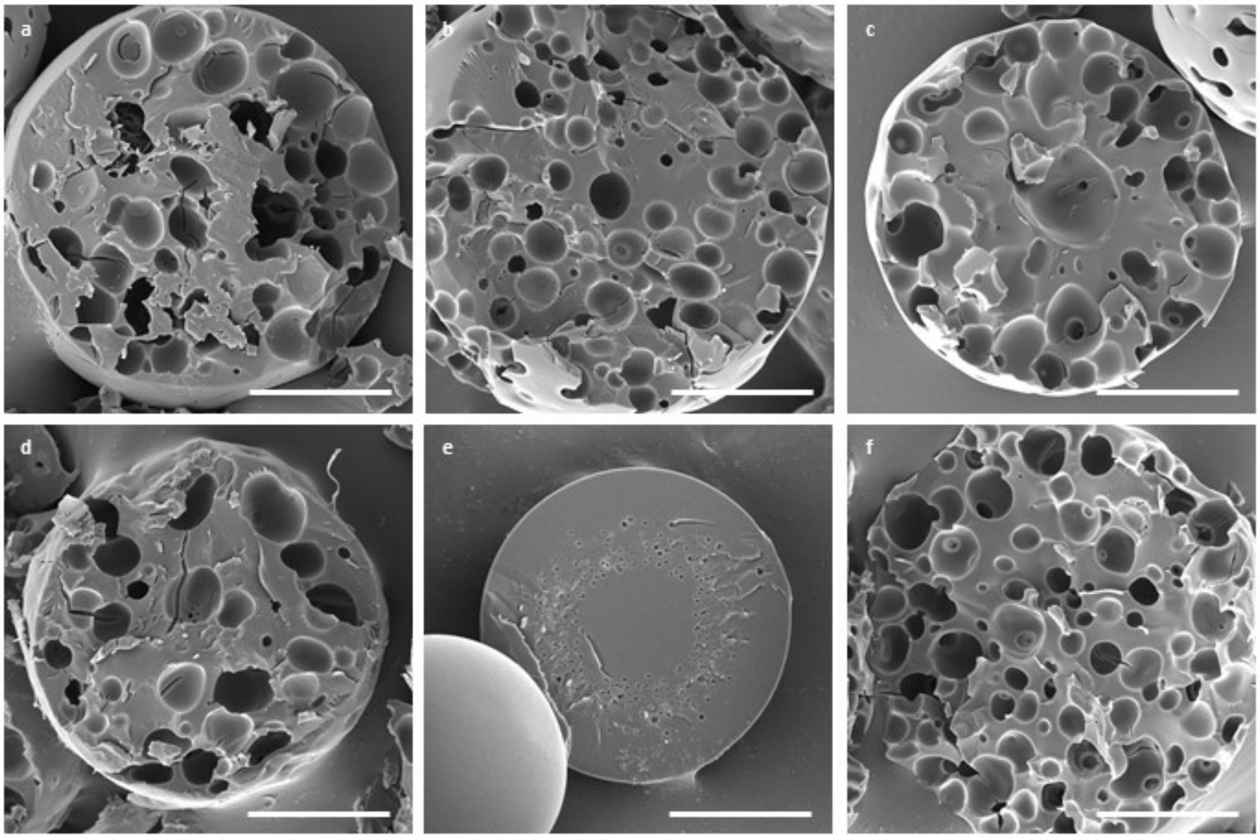

Supplementary Figure 2. Cross-sectional morphology of peptide loaded particles. Representative scanning electron micrographs of the cross-section of a) blank unloaded microsphere, b) leuprolide loaded, c) octreotide loaded, d) salmon calcitonin, e) dense leuprolide (no trehalose porosigen used in preformed microspheres), and f) bremelanotide loaded microspheres. (n > 3 independent experiments). Scale bar represents 20 μm.

Additionally, after incubation of blank microspheres with the peptide loading solutions there is a slight increase in particle size and glass transition temperature post hydration (Supplemental Table 1).

Supplementary Table 1. Physical properties of remote-loaded microspheres. Particle size distribution and glass transition temperature (Tg) of blank microspheres and peptide loaded microspheres measured with Mastersizer and Differential Scanning Calorimetry, respectively. (Mean ± SD, n = 3 independent experiments).

	Particle size and size distribution				Tg
	D (0.1)	D (0.5)	D (0.9)	Span	
Blank MS	33.0 ± 1.1	48.0 ± 0.8	63.6 ± 2.3	0.64 ± 0.06	45.4 ± 0.2
Leuprolide MS	36.3 ± 0.1	51.9 ± 0.1	68.64 ± 0.03	0.623 ± 0.002	49.1 ± 0.2
Octreotide MS	36.8 ± 0.1	51.74 ± 0.02	67.88 ± 0.01	0.602 ± 0.002	49.55 ± 0.04
Salmon Calcitonin MS	36.6 ± 0.1	54.5 ± 0.1	75.40 ± 0.06	0.712 ± 0.002	48.81 ± 0.01
Bremelanotide MS	45.8 ± 0.6	58.0 ± 0.6	73.3 ± 1.3	0.47 ± 0.03	48.11 ± 0.02

Additionally, peptide loading is comparable across several techniques for all peptides tested in porous microparticles (Supplementary Table 2).

Supplementary Table 2. Quantification of peptide loading by mass loss, extraction or nitrogen analysis.

Peptide	Sterilized	Microsphere concentration (mg/mL)	By mass loss (%)	By extraction (%)	By nitrogen analysis (%)
Leuprolide	No	180	9.64 ±0.01	9.37 ± 0.03	N/A
	Yes	180	9.48 ± 0.01	8.93 ± 0.13	N/A
	No	240	7.43 ± 0.01	7.57 ± 0.05	N/A
	Yes	240	7.25 ± 0.01	6.63 ± 0.10	N/A
Octreotide	No	180	8.49 ± 0.01	6.67 ± 0.10	6.79 ± 0.12
Vasopressin	No	180	8.00 ± 0.32	NA	8.49 ± 0.15
Salmon Calcitonin	No	180	8.17 ± 0.66	NA	10.9 ± 0.22
Exenatide	No	180	2.11 ± 0.47	NA	2.30 ± 0.20
Protirelin	No	180	2.77 ± 0.07	NA	2.77 ± 0.07
Pramlintide	No	180	NA	NA	9.77 ± 0.13
Bremelanotide	No	180	NA	8.37 ± 0.07	7.77 ± 0.11

Mean ± SEM (n = 2-3 independent experiments)
NA = quantification method not performed
Encapsulation efficiency (%) = peptide loading (%)/10%, as the theoretical loading was 10% w/w.

The level of burst release is important for leuprolide as there is a downregulation of the

gonadotropin receptor, as evidenced by the initial increase in testosterone in Fig. 3 and Supplementary Fig. 6. During *in vitro* studies Lupron Depot® microspheres exhibited an average initial burst of 22.3% release on day 1. We compared the initial burst release of leuprolide from irradiated and non-irradiated microspheres at both peptide loading concentrations (180 mg/mL and 240 mg/mL). We saw that microspheres loaded at the higher concentration had a lower initial burst independent of gamma irradiation exposure and all formulations had a very similar initial burst to that observed for the Lupron Depot® (Supplementary Fig. 3).

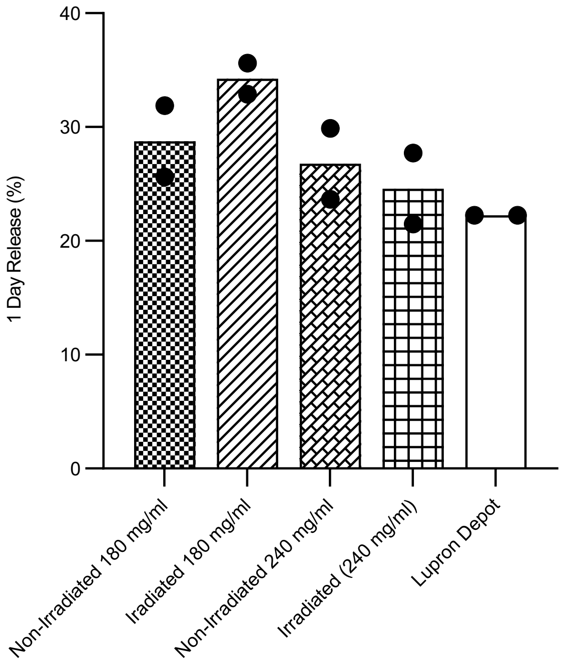

Supplementary Figure 3. Burst release of remote loaded microspheres compared to the commercial product. Initial burst release of leuprolide from irradiated and non-irradiated microspheres loaded at 180 mg/mL and 240 mg/mL was determined after 24 hours in PBST at 37 °C. Leuprolide acetate was remoted loaded in 0.5M HEPES buffer solution pH 7.4 and compared to Lupron Depot®. Data are presented as mean values (n = 2 independent experiments).

Looking closer at the *in vitro* release of leuprolide from microspheres loaded at 240 mg/mL with andwithout gamma irradiation we saw that the two formulations exhibited a similar release profile over 1 month (Supplementary Fig. 4).

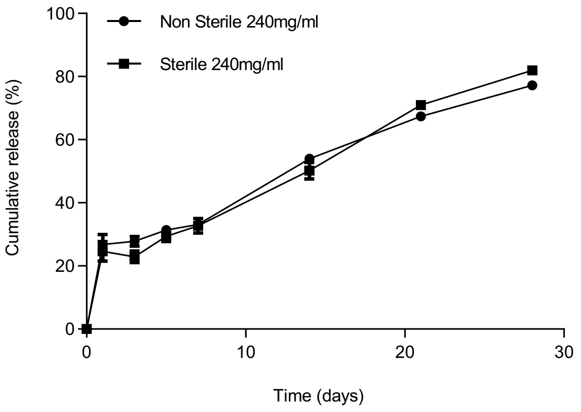

Supplementary Figure 4. Impact of gamma irradiation exposure on *in vitro* release of leuprolide from remote loaded microspheres. Cumulative *in vitro* leuprolide release was determined in PBST with mild agitation at 37 °C from remote-loaded PLGA microspheres with and without prior sterilization by gamma irradiation of the blank PLGA microspheres. Peptide loading concentration was from 240 mg/ml microsphere concentration. Data are presented as mean values ± SEM (n = 3 independent experiments)

In direct comparison of remote-loading to commercially used conventional encapsulation procedures for two important peptides, leuprolide and octreotide, we found remote loading to provide strongly similar or even favorable characteristics (Supplementary Tables 3 and 4). Remote loaded leuprolide using the same uncapped PLGA 75/25 (Wako, Inc.) to the 1-month Lupron Depot, the remote loading was highly efficient (e.g., 96.5%) relative to that reported for the commercial depot (103%)[29]. The remote-loaded and commercial formulations had respectively similar drug loading (9.37w/w% vs 8.95 w/w%), dry T_g (49.1 °C vs 48.6 °C), *in vitro* release and kinetics of testosterone reduction in rats (Fig. 3 and Supplementary Table 3). The largest difference was the size of the remote loaded formulation (52.1 μm vs 11.5 μm for the Lupron Depot®), which was also much more porous than the commercial formulation (see Supplementary Fig. 2 and Zhou et al.[28]). Additionally, gelatin was absent during remote loading like in the 3 and 6-month commercial Lupron Depot® formulations.[47]

Supplementary Table 3. Comparison of leuprolide remote-loaded PLGA microspheres to the commercial 1-month Lupron Depot®. (Mean ± SEM, n = 3 independent experiments).

	1-month Lupron Depot® [a]	Remote loaded leuprolide [c]
Volume weighted mean particle size	11.4 ± 0.5 µm [b]	52.14 ± 0.04 µm
PLGA type	75/25 14 kDa acid terminated [b]	75/25 14 kDa acid terminated
Drug loading (w/w)	8.95 ± 0.31% [b, d]	9.37 ± 0.03
Tg	48.6 ± 0.1°C [b]	49.1 ± 0.1
In vitro initial burst (%)	22.8 ± 0.4	28.8 ± 3.2
In vitro release behavior	Slow and continuous over 28 days	Slow and continuous over 28 days
In vivo efficacy behavior	Characteristic initial testosterone rise[e] followed by sustained chemical castration	Characteristic initial testosterone rise followed by sustained chemical castration

[a] The 1-month Lupron Depot® contains 1.5% w/w gelatin in the US.
[b] Values from the 1-month Lupron Depot® are from Zhou *et al.*[28].
[c] Data for standard porous formulation without gamma irradiation (180 mg/mL) before loading.
[d] Lupron Depot® contains 15% w/w mannitol on the particle surface.
[e] Initial rise occurs because of activation of gonadotropin receptor before its down regulation upon continuous binding to theleuprolide LHRH agonist.

For octreotide, as seen in Supplementary Table 4, besides a similar microsphere size and highly efficient loading for both remote loading and Sandostatin LAR®, the differences with remote loaded microspheres were more noticeable, beginning with the use of two different polymers, i.e., low molecular weight uncapped PLGA 75/25 (~14 kD) and glucose star ester terminated PLGA 55/45 (~52 kD)[30], respectively. Remote-loaded octreotide released the peptide *in vitro* continuously after a fairly low (~5.6% parent peptide) initial burst, whereas the commercial formulation exhibited an even lower burst release, followed by a lag time before continuous release (Fig. 2B). These release trends were mirrored in the differences in pharmacokinetics, which favored the remote-loaded formulation where no lag time in sustaining plasma levels was observed. Surprisingly, the Sandostatin LAR® exhibited a higher level of peptide acylation[14,30] than our formulation with the uncapped PLGA. Capping the polymer with an ester has been shown to reduce the acylation-triggered peptide-PLGA binding[14,53], although the end-capped glucose star polymer used in the Sandostatin LAR® rapidly forms linear acid terminated PLGA chains that bind octreotide[30].

Supplementary Table 4. Comparison of octreotide remote-loaded PLGA microspheres to the commercial 1-month Sandostatin LAR®. (Mean ± SEM, n = 3 independent experiments).

	1-month Sandostatin LAR®	Remote loaded octreotide
Volume weighted mean particle size	57.8 μm [a]	51.96 ± 0.03 μm
PLGA type	55/45 47 kDa star, glucose ester terminated [a]	75/25 14 kDa linear, acid terminated
Drug loading (%)	4.76 ± 0.11 [a, b]	6.67 ± 0.10
Tg	45.64 ± 0.06 [a]	49.55 ± 0.04
In vitro initial burst (%)	0.50 ± 0.01 [a]	5.58 ± 0.01
In vitro parent drug release behavior	2-week lag time before slow and continuous release over 6 weeks [a]	slow and continuous release > 8 weeks with low initial burst and nolag time
In vitro peptide acylationbehavior	Steady increase, reaching 72% by 6weeks [a]	Minimal (< 2.85% by 6 weeks)
In vivo pharmacokinetic behavior	Minimal exposure in first week before continuous exposure over 6weeks	Continuous exposure with no lagtime over 6 weeks

[a] Values from the 1-month Sandostatin LAR® are from Beig *et al.*[30].
[d] Sandostatin LAR® contains 16% w/w mannitol on the particle surface.

The general applicability of remote loading to additional peptides (exenatide, protirelin, pramlintide) was tested under the same loading conditions and at 180 mg/mL microsphere concentration as used for leuprolide. The titration of exenatide to PLGA (Supplementary Fig. 5) exhibited a step function, which is possibly due to the large c-window (i.e. the optimal range of experimental data values needed to maximize fit parameter confidence) at the current working condition.[54] Although the data have allowed us to estimate the binding enthalpy and the stoichiometry of the interaction between exenatide and PLGA, further work will be needed to optimize the c-window in order to obtain a more accurate binding affinity estimation. Remote loading of exenatide resulted in 23.1 ± 1.7 % encapsulation efficiency, with 2.3 ± 0.2 % loading (Supplementary Table 2). It is noteworthy that exenatide (pI = 4.38) was the only net negatively charged peptide at the remote loading condition (pH 7.4) among the tested peptides. Hence, it is logical to expect the repulsion between the negatively charged exenatide and the anionic polymer end groups limited binding, causing the relatively low loading and encapsulation efficiency of exenatide described above.

Similar to exenatide, protirelin also showed low encapsulation efficiency and loading in uncapped 75/25 PLGA microspheres by the remote loading process. For all peptides the loading was measured by several methods and the loading values obtained were fairly consistent regardless of

quantification method used (Supplementary Table 2).

Isothermal titration calorimetry results for protirelin supported the loading results as it did not exhibit significant evidence of binding, possibly due to the peptide's small size and lack of positively charged amino acid residues. The ionic state of the three amino acids-long protirelin relied solely on the ionization of the histidine residue with a pKa that is quite close to the pH of the remote loading condition. This weakly positive charge resulted in lack of charge-mediated interaction with the polymer end group. It is unknown why the registered heat of interaction of protirelin and polymer observed in the ITC study was endothermic, but it is important to note that the interaction between peptide and the polymer in DMSO is not the same as in the aqueous remote loading environment. Pramlintide has a steep initial curve for heats produced from peptide interaction with the polymer. This results in a high association constant (K_a = 0.346 μM^{-1}). Pramlintide also had a stoichiometry of almost 1.0 indicating that there is almost complete saturation of available acid terminations with peptide (Supplementary Fig. 5). The significant amount of heat released was strong evidence of binding between pramlintide and polymer carboxylic acid end groups. Pramlintide was positively charged at the remote loading condition, so it is reasonable to expect there were charge-mediated interactions between the peptide and the anionic polymer end group. The presence of this interaction is further supported by the high loading and encapsulation efficiency, 9.77 ± 0.13% and 97.8 ±1.3% respectively (Supplementary Table 2).

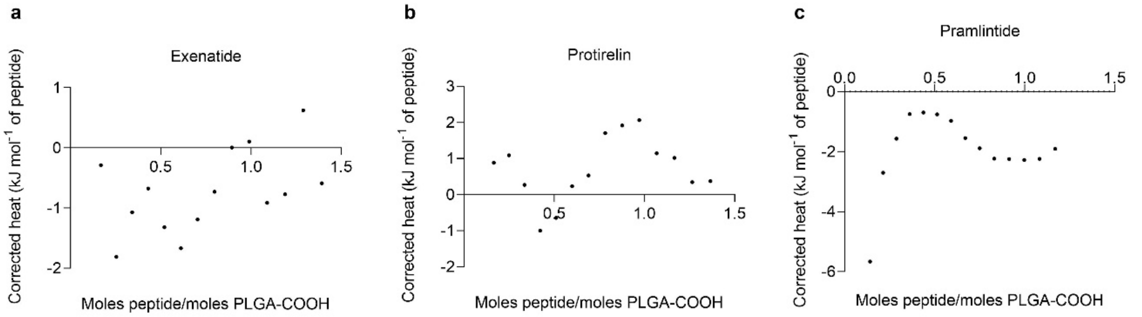

Supplementary Figure 5. Binding of additional peptides to PLGA. ITC thermograms of the binding affinity of exenatide (A), protirelin (B), and pramlintide (C) to 75/25 PLGA–COOH in DMSO was determined. Data shown as heat released per molar ratio of peptide to –COOH PLGA end group.

Octreotide, salmon calcitonin and bremelanotide all showed slow release during *in vitro* studies. In order to understand potential reasons for this slower release, their stability in phosphate buffer at refrigerated (4 ºC) and *in vitro* release conditions (37 ºC) for 1 or 2 weeks were tested. In 1 week at 37 ºC octreotide was most stable followed by bremelanotide then salmon calcitonin with ~95% peptide loss. After 2 weeks at 37 ºC salmon calcitonin was not detectable in phosphate buffer (Supplementary Table5). These stability results, particularly for salmon calcitonin, can partially explain the slower release as peptide likely degraded in the phosphate buffer once released as sampling was only done weekly. With more frequent sampling of release media, it is possible a higher peptide release would be detected.

Supplementary Table 5. Peptide stability in phosphate buffer. Mean ± SEM (n=3 independent experiments).

Stability condition/ timepoint	Octreotide (µg/mL)	Bremelanotide (µg/mL)	Salmon Calcitonin (µg/mL)
Day 0	59.4 ± 0.04	62.91 ± 0.16	61.98 ± 0.34
4 ºC/ Day 7	59.6 ± 0.11	57.67 ± 0.01	61.4 ± 0.24
37 ºC/ Day 7	55.55 ± 0.16	53.53 ± 0.03	2.97 ± 0.03
37 ºC/ Day 14	49.63 ± 0.56	50.77 ± 0.25	Not Detected

In vivo rat studies with control microspheres (without gamma irradiation exposure) loaded at 180 mg/mL and 240 mg/mL leuprolide concentration resulted in castration levels within one-week post administration (Supplementary Fig. 6) as was seen with irradiated microspheres and Lupron Depot® (Fig. 3). These results support the reproducibility of the simple remote loading method to provide sustained peptide release *in vivo*.

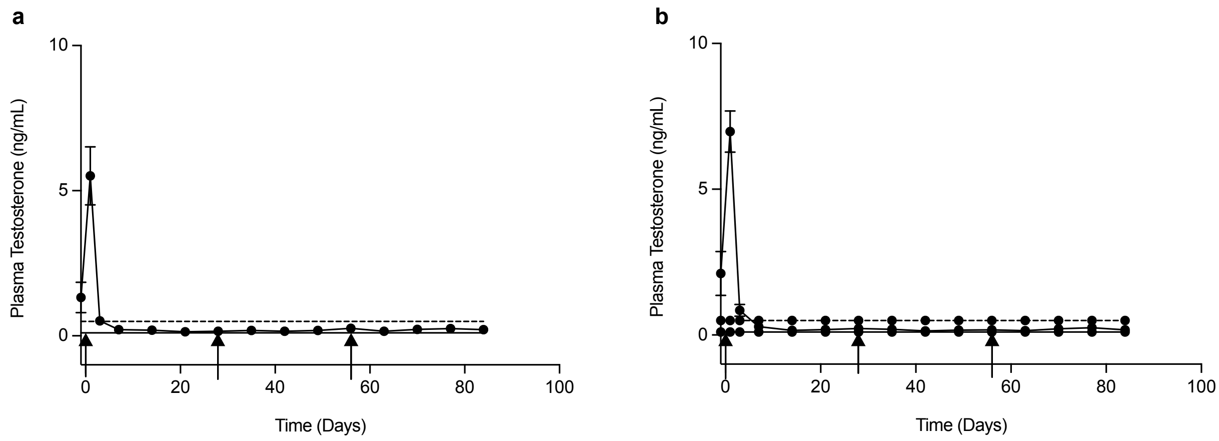

Supplementary Figure 6. Pharmacokinetic response after administration of non-sterilized microspheres. In vivo plasma testosterone levels of male Sprague-Dawley rats subcutaneously administered 3 monthly doses of 180 mg/mL (A) and 240 mg/mL (B) leuprolide loaded 75/25 PLGA microspheres. Microspheres were not exposed to gamma irradiation. Dashed line indicates castration level (0.5 ng/mL). Dotted line indicates analysis limit of detection (0.1 ng/mL). Data are presented as mean values ± SEM (n = 6 rats). Arrow indicates days of dosing.

In vitro release of microspheres loaded with bremelanotide are described in Supplementary Fig. 7**.** Initial burst release was slightly higher (~40%) than leuprolide (Fig. 2A) followed by continuous release for more than 1 month.

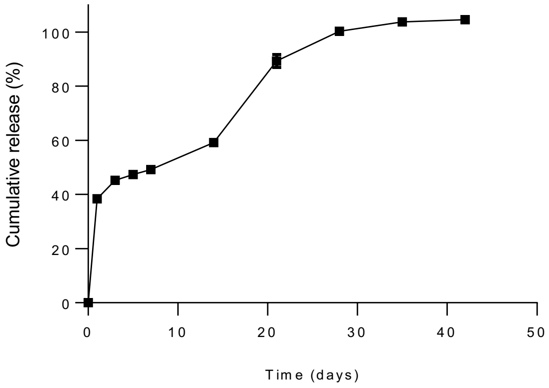

Supplementary Figure 7. Cumulative in vitro release of bremelanotide from remote loaded PLGA microspheres. Drug loading was 83.7% ± 0.7% w/w. Data are presented as mean values ± SEM (n = 3 independent experiments).

To determine if the initial burst release of the remote-loaded microspheres could be reduced, we investigated two approaches based on adjusting the polymer purity and porosity. First, to adjust polymer purity, water soluble acids of polymers were removed according to a patented method.[55] We dissolved 5 g of polymer in 10 mL methylene chloride and the solution was poured into 500 mL of hot water at about 60 ºC with stirring. When the methylene chloride gradually evaporated, purified polymer floating on the surface could be collected and then dried under reduced pressure for next use. Second, we prepared a nonporous (or dense) formulation from both unpurified and purified PLGA, which was created by removing trehalose from the inner water phase of the preformed microspheres. The four microsphere formulations (+/- purification, +/- pore forming trehalose) were loaded with leuprolide by the normal procedure at 180 mg/mL microspheres. As seen in in Supplementary Table 6, the loading varied little between 8-9 wt% (or 80-90% encapsulation efficiency). The different inner structures between porous and dense remote-loaded leuprolide microspheres can be seen in Supplementary Fig. 2B and Supplementary Fig. 2E, respectively. As shown in Supplementary Fig. 8, the decrease in porosity showed a substantial decrease in initial burst (~40%). However, purification of the polymer showed little or no initial burst reduction, indicating relative purity of the polymer used for remote loading data discussed earlier. Note that the slightly lower loading and initial burst values in the control porous and non-purified

relative to those described above may have resulted from a different batch of PLGA used.

Supplementary Table 6. Comparison of loading of leuprolide in preformed PLGA microspheres fabricated with or without trehalose or added PLGA purification steps. Theoretical load was 10 w/w%. Mean ± SEM (n = 3 independent experiments).

Preformed microspheres	Loading (%)
Porous, nonpurified	8.0 ± 0.3
Dense, nonpurified	8.3 ± 0.1
Porous. purified	9.0 ± 0.3
Dense, Purified	8.4 ± 0.3

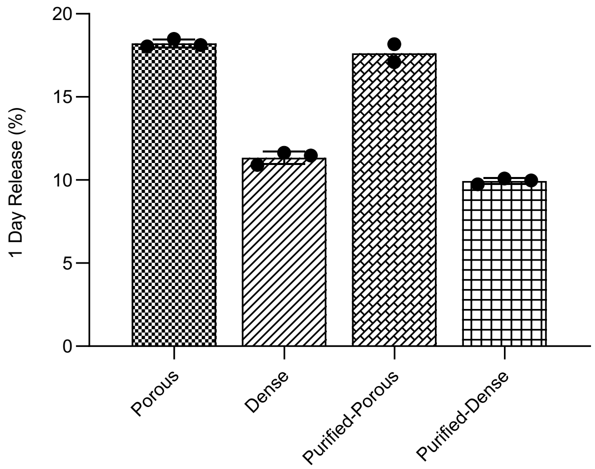

Supplementary Figure 8. Impact of polymer purity and particle formulation on initial burst release of leuprolide. *In vitro* initial burst release of leuprolide from microspheres fabricated with or without trehalose (i.e., porous or dense microspheres) or PLGA purification steps. Data are presented as mean values for purified-porous group (n = 2 independent experiments), and as mean values ± SEM for porous, dense and purified-dense groups (n = 3 independent experiments).

Supplementary References

51. Göpferich, A. Mechanisms of polymer degradation and erosion. Biomaterials 17, 103- 114 (1996).

52. Davidson, J. A. Mercury porosimetry studies II. The application of mercury porosimetry to porous polymer powders. Powder Technol 23, 239-244 (1979).

53. Sophocleous, A. M., Zhang, Y. & Schwendeman, S. P. A new class of inhibitors of peptide sorption and acylation in PLGA. J Control Release 137, 179-184 (2009).

54. Hansen, L. D., Fellingham, G. W. & Russell, D. J. Simultaneous determination of equilibrium constants and enthalpy changes by titration calorimetry: Methods, instruments, and uncertainties. Anal Biochem 409, 220-229 (2011).

55. Yamamoto M., Osaka, H., Ogawa Y. & Miyagawa T. Polymer, production and use thereof. US 4728721 (1988).
