# Supplementary material for: Efficient aqueous remote loading of peptides in poly(lactic-co-glycolic acid)
Source: Nat Commun. 2022 Jun 8;13:3282. doi: 10.1038/s41467-022-30813-7 (PMC9177552; doi:10.1038/s41467-022-30813-7)
Supplement: Supplementary file 2 — Reporting Summary [file 41467_2022_30813_MOESM2_ESM.pdf]

nature portfolio

Corresponding author(s): Steven P. Schwendeman

Last updated by author(s): Apr 13, 2022

Reporting Summary

Nature Portfolio wishes to improve the reproducibility of the work that we publish. This form provides structure for consistency and transparency in reporting. For further information on Nature Portfolio policies, see our Editorial Policies and the Editorial Policy Checklist.

Statistics

For all statistical analyses, confirm that the following items are present in the figure legend, table legend, main text, or Methods section.

n/a	Confirmed	
☐	☒	The exact sample size (n) for each experimental group/condition, given as a discrete number and unit of measurement
☐	☒	A statement on whether measurements were taken from distinct samples or whether the same sample was measured repeatedly
☐	☒	The statistical test(s) used AND whether they are one- or two-sided<br>*Only common tests should be described solely by name; describe more complex techniques in the Methods section.*
☒	☐	A description of all covariates tested
☐	☒	A description of any assumptions or corrections, such as tests of normality and adjustment for multiple comparisons
☐	☒	A full description of the statistical parameters including central tendency (e.g. means) or other basic estimates (e.g. regression coefficient) AND variation (e.g. standard deviation) or associated estimates of uncertainty (e.g. confidence intervals)
☐	☒	For null hypothesis testing, the test statistic (e.g. F, t, r) with confidence intervals, effect sizes, degrees of freedom and P value noted<br>*Give P values as exact values whenever suitable.*
☒	☐	For Bayesian analysis, information on the choice of priors and Markov chain Monte Carlo settings
☒	☐	For hierarchical and complex designs, identification of the appropriate level for tests and full reporting of outcomes
☒	☐	Estimates of effect sizes (e.g. Cohen's d, Pearson's r), indicating how they were calculated

Our web collection on statistics for biologists contains articles on many of the points above.

Software and code

Policy information about availability of computer code

Data collection	Raw HPLC/UPLC data was collected and processed using Empower (version 3). ITC data was collected and processed using NanoAnalyze (version 3.7.5). Non-compartment PK analysis was done using Matlab (version 2020b)
Data analysis	Data presented in this study were analyzed using GraphPad Prism, Microsoft Excel and MATLAB

For manuscripts utilizing custom algorithms or software that are central to the research but not yet described in published literature, software must be made available to editors and reviewers. We strongly encourage code deposition in a community repository (e.g. GitHub). See the Nature Portfolio guidelines for submitting code & software for further information.

Data

Policy information about availability of data

All manuscripts must include a data availability statement. This statement should provide the following information, where applicable:

- Accession codes, unique identifiers, or web links for publicly available datasets
- A description of any restrictions on data availability
- For clinical datasets or third party data, please ensure that the statement adheres to our policy

The authors declare that all data supporting the results in the study are available within the paper and its supplementary information. Additionally, data is available on FigShare (https://figshare.com/account/home#/projects/81131) and can be provided upon request.

Field-specific reporting

Please select the one below that is the best fit for your research. If you are not sure, read the appropriate sections before making your selection.

[x] Life sciences [ ] Behavioural & social sciences [ ] Ecological, evolutionary & environmental sciences

For a reference copy of the document with all sections, see nature.com/documents/nr-reporting-summary-flat.pdf

Life sciences study design

All studies must disclose on these points even when the disclosure is negative.

Sample size	Sample size in vitro release testing was set to n=2 or n=3. Variation in these in vitro assay is typically very low. Sample size for in vivo testing in rats was selected as n=4-6 to allow for any variations between animals in terms of PK response as well as if there were any challenges in collecting time points. Sample sizes for in vitro and in vivo studies were chosen on the basis of preliminary data from pilot experiments and previously reported experiments with similar formulations. Statistical significance of t-test of the AUCs was assumed $p < 0.05$.
Data exclusions	No data was excluded from analysis.
Replication	Remote loading studies for each peptide were done in duplicate or triplicate to ensure reproducibility of loading and to ensure enough particles were available for subsequent in vitro studies. Loading was also measured by multiple techniques to confirm values. Loading was repeated to prepare particles for in vivo studies and loading values compared to earlier data to ensure reproducibility within variability. Replication of loading was successful.
Randomization	Randomization was not relevant to the studies in this work as specific polymer formulations were made at discrete peptide concentrations and tested for release in vitro and in vivo. Tests regarding peptide binding to polymer was not randomized as the polymer was kept consistent between formulations so as to determine peptide binding to the specific polymer by this remote loading technique.
Blinding	Investigators were not blinded to group allocation during the experiments and outcome assessment because the data analyses in this study were based on objectively measurable data.

Reporting for specific materials, systems and methods

We require information from authors about some types of materials, experimental systems and methods used in many studies. Here, indicate whether each material, system or method listed is relevant to your study. If you are not sure if a list item applies to your research, read the appropriate section before selecting a response.

Materials & experimental systems

n/a	Involved in the study	
[x]	[ ]	Antibodies
[x]	[ ]	Eukaryotic cell lines
[x]	[ ]	Palaeontology and archaeology
[ ]	[x]	Animals and other organisms
[x]	[ ]	Human research participants
[x]	[ ]	Clinical data
[x]	[ ]	Dual use research of concern

Methods

n/a	Involved in the study	
[x]	[ ]	ChIP-seq
[x]	[ ]	Flow cytometry
[x]	[ ]	MRI-based neuroimaging

Animals and other organisms

Policy information about studies involving animals; ARRIVE guidelines recommended for reporting animal research

Laboratory animals	Rats, Sprauge Dawley, male, 7 weeks old
Wild animals	No wild animals were used in the study
Field-collected samples	No field collected samples were used in the study
Ethics oversight	Animal studies are conducted according to University of Michigan Committee on Use and Care of Animals (IACUC) protocol PRO00007890 and PRO00009970

Note that full information on the approval of the study protocol must also be provided in the manuscript.
